# Connectivity of EEG synchronization networks increases for Parkinson’s disease patients with freezing of gait

**DOI:** 10.1038/s42003-021-02544-w

**Published:** 2021-08-30

**Authors:** Eitan E. Asher, Meir Plotnik, Moritz Günther, Shay Moshel, Orr Levy, Shlomo Havlin, Jan W. Kantelhardt, Ronny P. Bartsch

**Affiliations:** 1 Department of Physics, Bar-Ilan University, Ramat-Gan, Israel; 2 Center of Advanced Technologies in Rehabilitation, Sheba Medical Center, Ramat Gan, Israel; 3 Department of Physiology and Pharmacology, Sackler Faculty of Medicine, Tel Aviv University, Tel Aviv, Israel; 4 Sagol School of Neuroscience, Tel Aviv University, Tel Aviv, Israel; 5 Nuclear Research Center Negev, Beer-Sheva, Israel; 6 Institute of Physics, Martin-Luther-University Halle-Wittenberg, Halle, Germany

**Keywords:** Parkinson's disease, Parkinson's disease

## Abstract

Freezing of gait (FoG), a paroxysmal gait disturbance commonly experienced by patients with Parkinson’s disease (PD), is characterized by sudden episodes of inability to generate effective forward stepping. Recent studies have shown an increase in beta frequency of local-field potentials in the basal-ganglia during FoG, however, comprehensive research on the synchronization between different brain locations and frequency bands in PD patients is scarce. Here, by developing tools based on network science and non-linear dynamics, we analyze synchronization networks of electroencephalography (EEG) brain waves of three PD patient groups with different FoG severity. We find higher EEG amplitude synchronization (stronger network links) between different brain locations as PD and FoG severity increase. These results are consistent across frequency bands (theta, alpha, beta, gamma) and independent of the specific motor task (walking, still standing, hand tapping) suggesting that an increase in severity of PD and FoG is associated with stronger EEG networks over a broad range of brain frequencies. This observation of a direct relationship of PD/FoG severity with overall EEG synchronization together with our proposed EEG synchronization network approach may be used for evaluating FoG propensity and help to gain further insight into PD and the pathophysiology leading to FoG.

## Abstract (web summary)

Asher et al analyzed synchronization networks of electroencephalography brain waves from three Parkinson’s disease (PD) patient groups with different freezing of gait (FoG) severity using tools based on network science and non-linear dynamics. Their findings suggest that there is a potential relationship between PD/FoG severity and overall EEG synchronization and their approach could be used to help gain further insight into the pathophysiology leading to FoG.

## Introduction

Parkinson’s disease (PD) is a progressive neurodegenerative disorder with numerous non-motor and motor-related symptoms including tremor at rest, rigidity, akinesia (or bradykinesia), and postural instability^1^. In addition, freezing of gait (FoG)—a form of akinesia that manifests itself by the inability to generate effective forward stepping—is common among patients in the advanced stages of the disease^2^. FoG is a significant risk factor for falls and injuries^3^ and, therefore, is considered one of the most disabling symptoms of PD having severe consequences to the patients’ quality of life. It is important to note that freezing is not a universal PD phenomenon as it occurs in just about half of PD patients^4,5^ and is less frequent in women and patients with pronounced tremor^6^.

Previous research on PD-related movement disorders has focused on the analysis of data characterizing gait and limb dynamics or locomotion^7–11^, as well as electromyography (EMG) activation of lower limb muscles^3^. Additionally, in the context of freezing, FoG-related changes in physiological signals such as electrocardiography^12^, galvanic skin response^13^, and electroencephalography (EEG)^14–17^) have been reported. These signals, which measure certain cortical, mental, spinal, motor, and autonomic nervous system functions presumably interact with each other as a network to generate physiologic function^18–20^. A few particular network interactions have recently been studied in PD patients. For example, it has been shown that EMG-EEG coupling increases at the beginning of intentional stops and FoG episodes^21^, and that during locomotion, EEG synchronization between brain hemispheres is significantly higher in PD patients compared to elderly controls^22^.

Functional interactions in the brain became a main field of interdisciplinary research in recent years^23^, and brain networks have been identified based on various signals obtained from, for example, magnetic resonance imaging (MRI), positron emission tomography (PET), magnetoencephalography (MEG) and EEG^24^. By applying methods of modern network science, the normal brain has been characterized as a hierarchical, modular network with high clustering, short path length, and a ‘backbone’ of highly connected network nodes (“hubs”)^24,25^. Consequently, deviations from the “normal brain network” have been associated with disease and neurological disorders^26^, yet, findings regarding basic network properties remain controversial^24^. This may be in part due to the different signals and methods of analysis used in the studies and whether weighted or unweighted networks were considered. Another limiting factor is the lack of a generally accepted approach for defining thresholds and applying surrogate and normalization techniques to control for spurious network links that do not reflect real interactions.

In this paper, we develop a new network approach based on synchronization and cross-modulation analysis that can systematically distinguish between significant and non-significant interactions in brain activity. While there are a few studies on brain networks in PD based on fMRI and MEG data, which have been performed during a specific motor task (e.g., finger tapping) or resting state^27–29^, our work is first in studying EEG synchronization networks during locomotion. Up until recently, such research was hampered by intrinsic movement artifacts in the EEG data but we and others have developed methods to remove any such non-physiological information (see, e.g., ^30,31^).

Among the first studies of EEG brain dynamics during FoG has been the work by Shine et al.^14^, who reported higher theta band power and theta frequency coupling within frontal and central electrodes while transitioning from normal walking to FoG during up-and-go tasks. In follow-up studies by the same group, Handojoseno et al.^15^ have shown that also beta and gamma coupling increases during FoG, and that for turn-triggered FoG, beta and theta power is enlarged predominantly in the occipital and parietal areas^16,17^. Based on these findings, in our present study, we hypothesize that PD+FoG patients have higher EEG coupling also during normal walking as compared to elderly controls and PD-FoG patients, and that increased EEG connectivity is observed across all physiologically-relevant frequency bands. In order to measure EEG connectivity among different channels, we apply phase synchronization analysis, which is a well-established method to quantify interactions between complex dynamical systems with oscillating behavior^32–34^, and it has been applied to identify coupling between various physiological systems^9,35–42^. However, various factors related to the properties of the analyzed signals, external and intrinsic noise, and data pre-processing procedures can lead to spurious detection of phase synchronization^43^. In order to discriminate between such spurious network links and real network interactions, we introduce a novel significance measure that weights maximum synchronization against background noise.

Applying our method on multi-channel EEG data recorded from different groups of PD patients and healthy elderly controls during overground walking experiments, we construct brain networks using the property of network synchronizability, where link strengths directly correspond to the degree of phase synchronization between brain-wave amplitudes from different network nodes (i.e., EEG channels or brain lobes)^41^. Investigating systematically all links in the brain, we show that brain networks are most pronounced, on the level of the whole cortex, in PD patients with the highest disease severity. This increased synchronization is independent of EEG frequency as we find it to be consistent across all physiologically relevant brain waves. Of particular interest is our result that PD+FoG patients, who did not show freezing of gait during our experiments (therefore labeled ‘PD+FoG^−^’), have significantly lower brain network synchronization than PD+FoG patients who did show FoG episodes during the experiments (‘PD+FoG^+^’ patients). Moreover, since EEG synchronization networks of PD+FoG^−^ patients are more similar to non-freezers (‘PD-FoG’), our results suggest that FoG propensity may change on a daily/hourly basis. Our approach may thus be applicable to PD monitoring and treatment selection, as well as can help in evaluating the severeness of the disease.

## Results

**Fig. 1:**
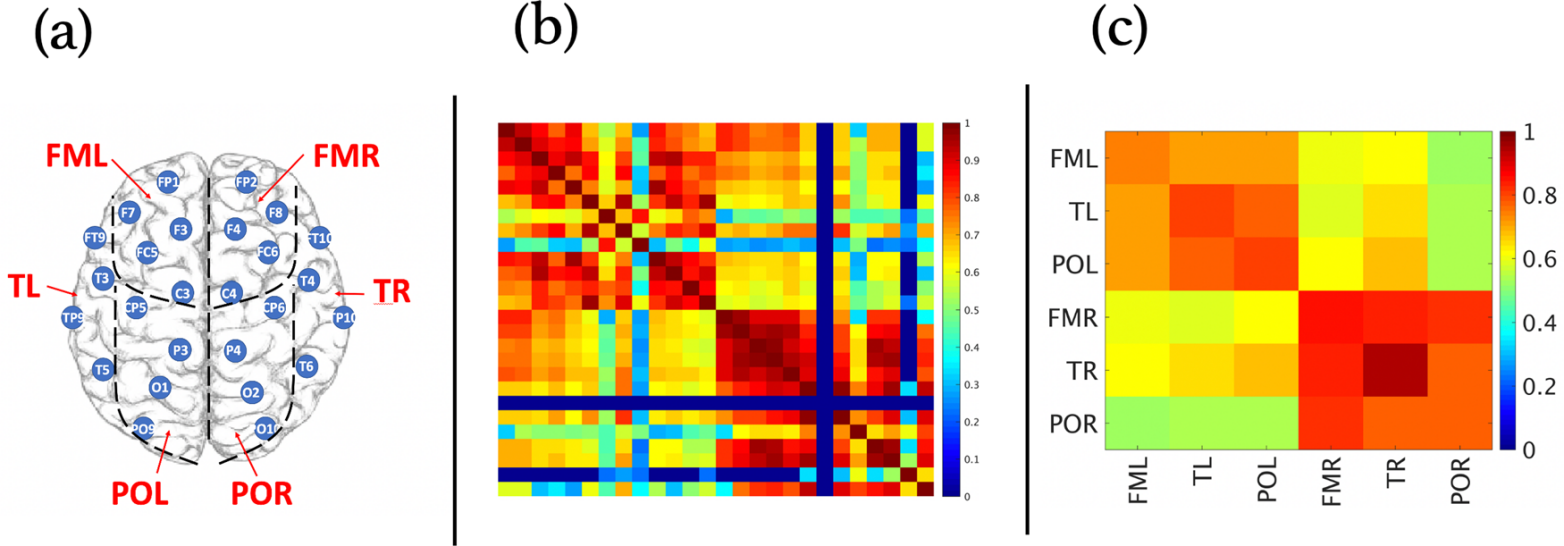
Construction of brain wave synchronization matrices based on EEG electrode position. **a** Data were recorded by a 32-channel EEG montage according to the international 10−20 standard system (the four midline electrodes Fz, Cz, Pz, Oz, and the two reference electrodes M1 and M2 were excluded from the analysis). Electrodes were grouped according to different brain lobes (as indicated by the dashed lines): frontal motor left—FML (including electrodes FP1, F7, F3, FC5, and C3); frontal motor right—FMR (FP2, F8, F4, FC6, and C4); temporal left—TL (FT9, T3, TP9, and T5); temporal right—TR (FT10, T4, TP10, and T6); parietal occipital left—POL (CP5, P3, O1, and PO9); and parietal occipital right—POR (CP6, P4, O2, and PO10). **b** Matrix of the averaged synchronization indexes ${\langle {R}^{{j}_{1},{j}_{2}}\rangle }_{\nu }$ for all combinations of *α*-amplitude signals *j*_1_ and *j*_2_ from all 26 electrodes of a single PD+FoG^+^ subject. Averaging was done over all normal walking segments *ν*. Note that we exclude electrodes with high impedance or high standard deviations from our analysis (e.g., the two dark blue lines in panel (**b**) corresponding to electrode CP6). **c** The matrix elements of panel (**b**) are averaged according to the definition of brain lobes shown in (**a**) to obtain a brain wave synchronization matrix. Matrix elements that correspond to the same electrode interaction (i.e., the diagonal elements in (**b**)) have been excluded from the average.

**Fig. 2:**
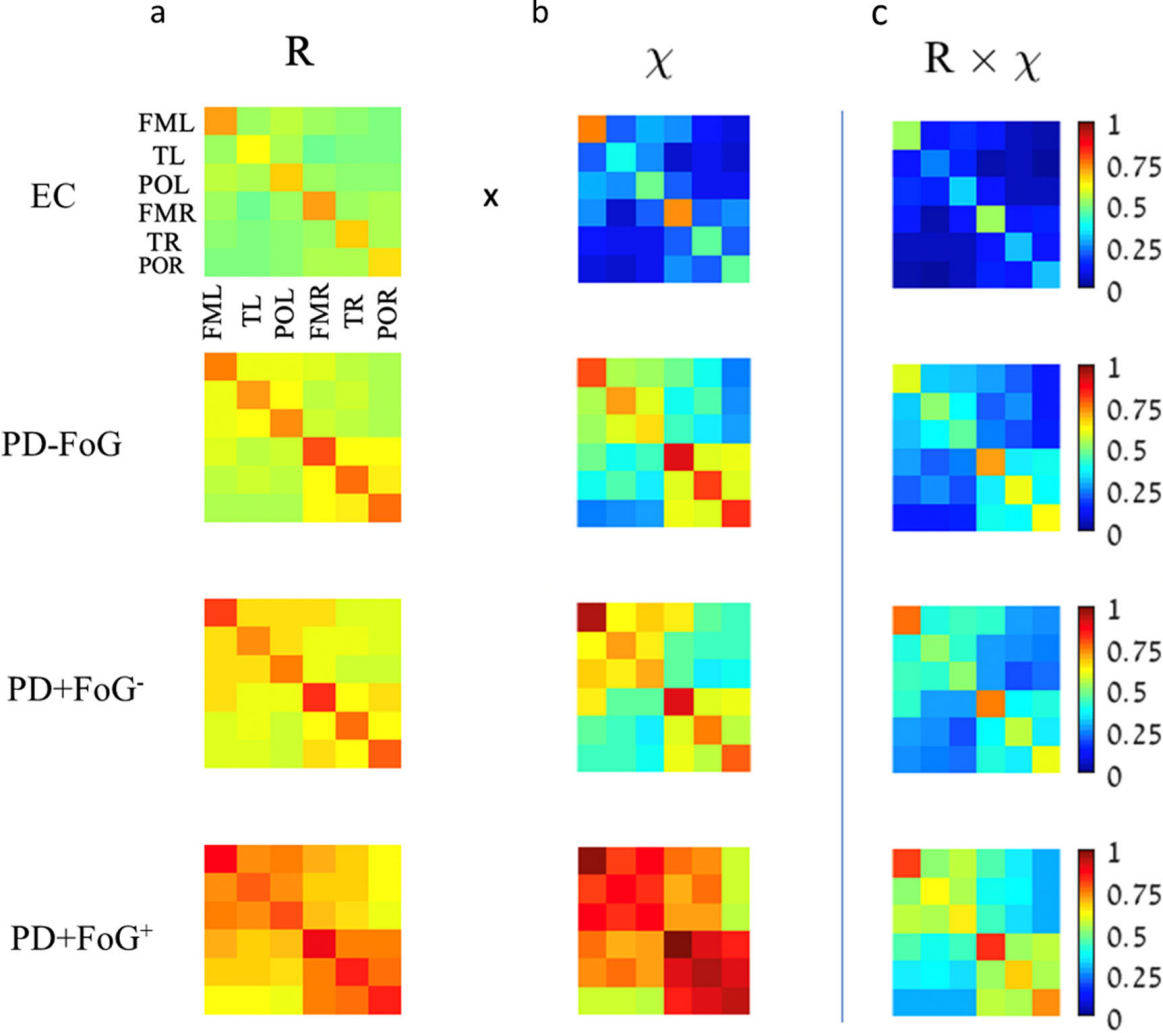
Brain lobe interaction matrix calculated from synchronization and fraction matrix. Element-wise multiplication of **a** synchronization matrix **R** and **b** fraction matrix ***χ*** yields **c** the total brain lobe interaction matrix **R** × ***χ*** that is used as the adjacency matrix of the underlying physiological network of brain lobe interactions (Fig. 3). In this figure, all matrices are derived for *α*−*α* interactions during normal walking epochs. Group average matrices for EC, PD-FoG, PD+FoG^−^ and PD+FoG^+^ (from top to bottom) are shown. Note, there is a dramatic increase in brain lobe interaction with the severity of Parkinson’s disease which is represented by (i) higher levels of *α*−*α* synchronization, as well as (ii) higher fractions of significant interactions.

We calculate two kinds of interaction matrices based on (i) the synchronization index *R* between all combinations of instantaneous amplitude signals *j*_1_ and *j*_2_ (see Figs. 1 and 2a for *α*−*α* interaction), and (ii) the fraction of significant vs. non-significant interactions (“links”) based on the detected τ^*^ and *W* values (Fig. 2(b)). More specifically, for the “fraction” matrix ***χ*** we set the matrix element ${\chi }_{{j}_{1},{j}_{2}}=1$, if the corresponding *j*_1_ − *j*_2_ interaction in a given segment of length *L* is significant (i.e., τ^*^ ∈ [−0.05, 0.05] seconds and *W* > 2.5), otherwise ${\chi }_{{j}_{1},{j}_{2}}=0$. The averaging across all normal walking segments *ν* and across the brain lobes is performed in the same way as for the synchronization matrix **R** (cp. Fig. 1). The resulting **R** and ***χ*** matrices for each subject are then multiplied element-wise to obtain the total brain lobe interaction matrix **R** × ***χ*** (Fig. 2c).

**Fig. 3:**
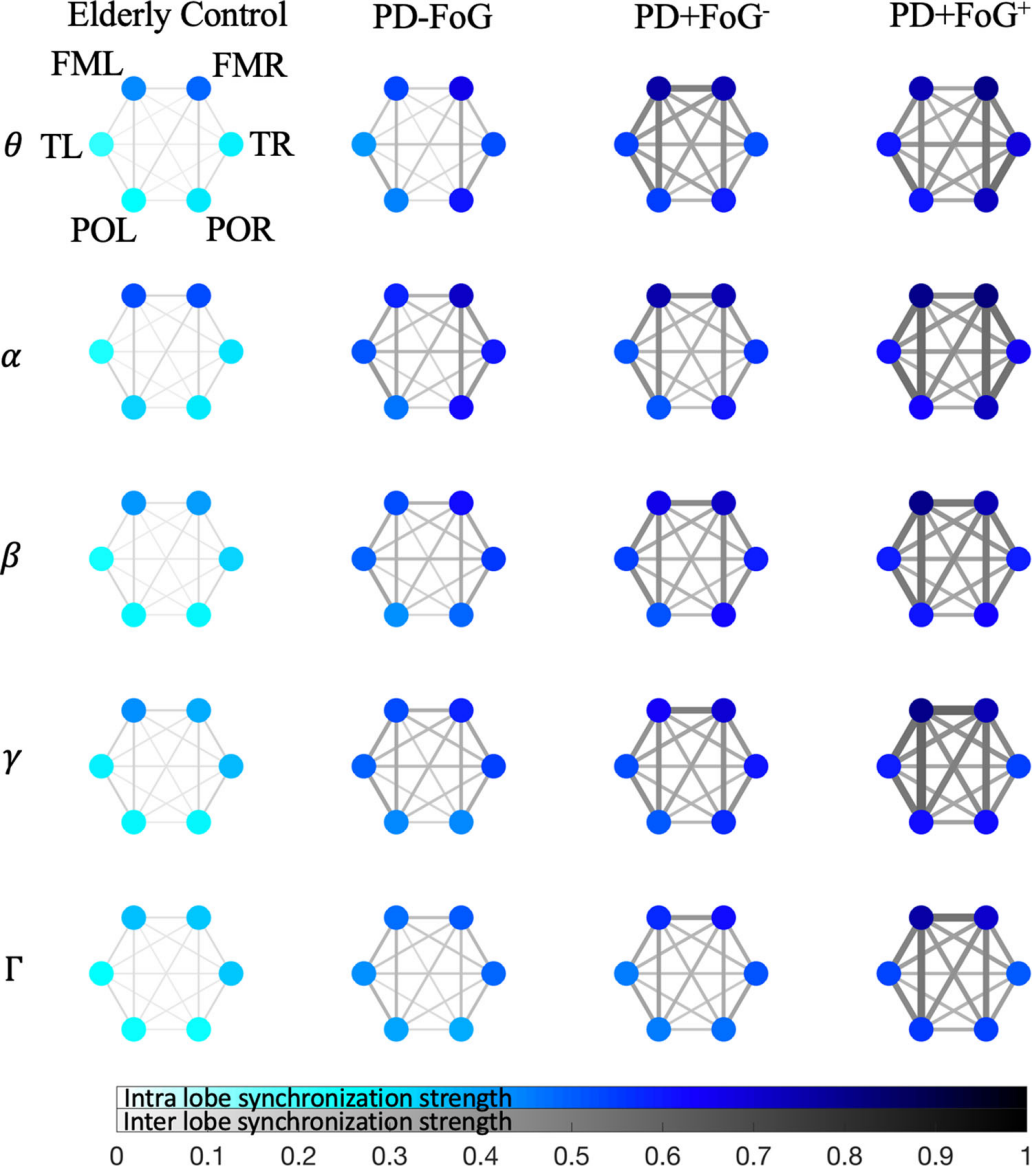
Physiological networks of brain lobe interactions for different EEG frequency bands. The brain lobe interaction matrices **R** × ***χ*** are used to construct physiological networks for each frequency band and for each group during normal walking (cp. Fig. 2c for *α*−*α* interactions for all four groups). Network nodes correspond to the six brain lobes and the color-coding of the nodes is according to the intra-lobe interaction values obtained from the diagonal matrix elements of the lobe-averaged **R** × ***χ*** matrices. Weighted network links reflect inter-lobe interaction as given by the value of the non-diagonal matrix elements, and darker gray color and thicker lines represent stronger interactions. Subjects with Parkinson’s disease (PD) generally exhibit higher levels of brain lobe interactions, and the highest values are observed for PD+FoG^+^ consistently across all EEG frequency bands.

The brain lobe interaction matrix represents an adjacency matrix of the underlying physiological network with brain lobes as network nodes and the matrix elements as weighted network links. We obtain these interaction matrices and networks separately for each of the five defined frequency bands (excluding the *δ* band), adjusting the segment length *L* accordingly (see Table 1). The resulting brain lobe interaction matrices are averaged over all the participants in each of the four study groups EC, PD-FoG, PD+FoG^−^, PD+FoG^+^ to construct their group-averaged networks for all frequency bands (Fig. 3).

**Table 1:** EEG frequency bands and number of analyzed segments for each group and frequency band.

EEG band	frequency [Hz]	L [sec]	EC	PD-FoG	PD+FoG^−^	PD+FoG^+^
*δ*	0.5−3.99	15	63	36	11	16
*θ*	4−7.79	5	445	291	119	193
*α*	7.8−15.59	3	819	538	235	399
*β*	15.6−31.19	1.5	1764	1182	510	903
*γ*	40−62.39	1	2726	1805	783	1397
Γ	62.4−90	0.5	5567	3710	1611	2897

The segment length *L* in which the synchronization index *R* is calculated depends on the analyzed EEG frequency band. *L* is chosen so that about 10−15 amplitude oscillations are present in each window. Naturally, the number of available windows increases with higher frequencies. For our analyses, we select only windows recorded during normal walking that do not contain stops, FoG episodes, or FoG triggers. We excluded the *δ*-band interactions from the following analyses because of insufficient statistics (<100 available segments).

**Fig. 4:**
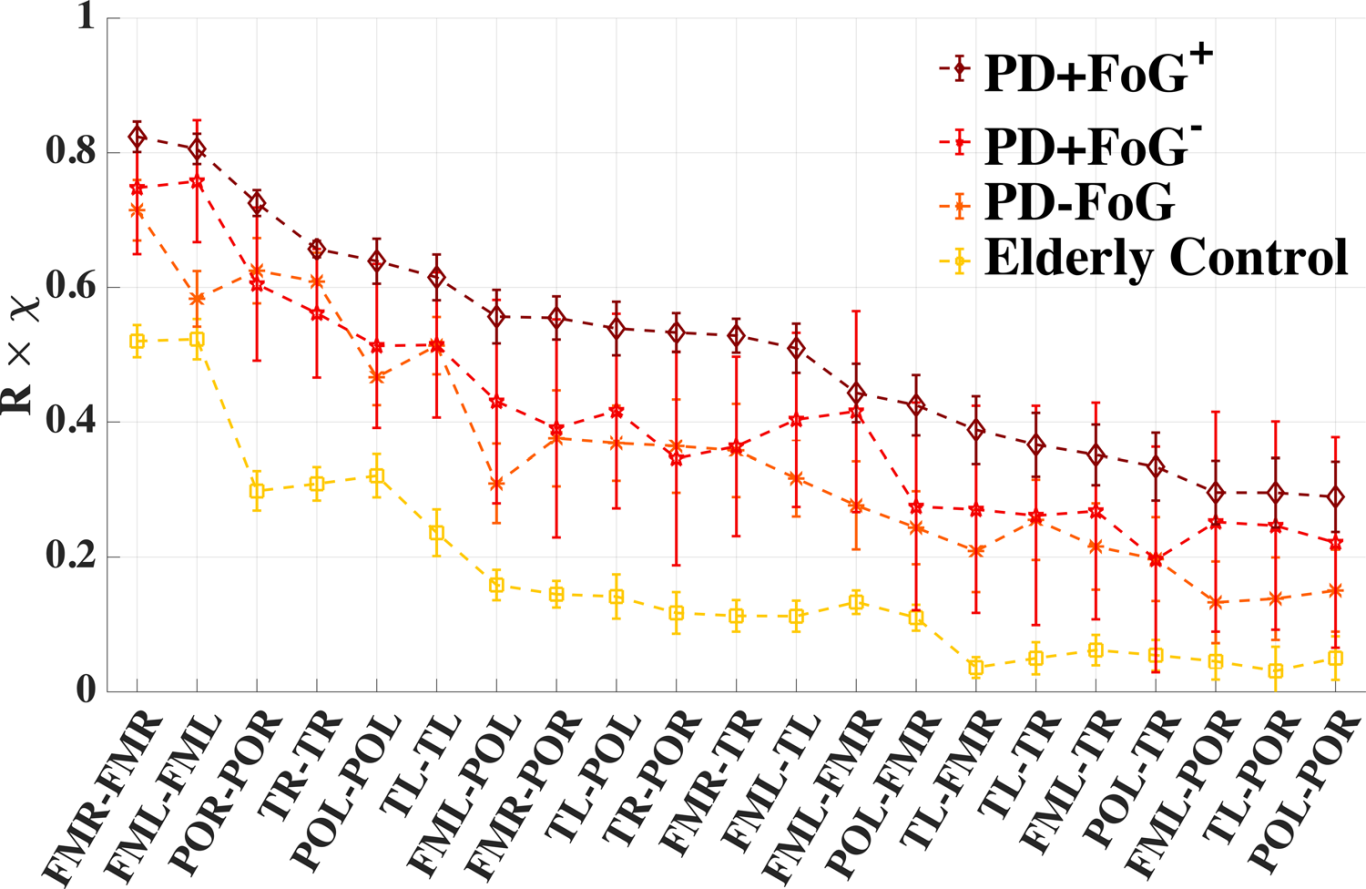
Rank distributions for the strength of brain lobe interactions. Group-averaged values of individual brain lobe *α*−*α* interactions (i.e., 21 matrix elements of the upper triangular part of the matrices in Fig. 2c) for the different groups of subjects. The ranking follows the values of the PD+FoG^+^ group. Ranks 1 and 2 correspond to interactions within the frontal motor areas (FMR−FMR and FML−FML) that are strongest for all groups. Note that values of each **R** × ***χ*** matrix element are consistently highest for PD+FoG^+^ and lowest for EC, with PD-FoG and PD+FoG^−^ falling in-between. Symbols and error bars represent the group means and standard error, respectively. Error bars have been calculated using a bootstrap method^70^.

Our results for the *α* band shown in Fig. 2 demonstrate that PD patients show stronger brain lobe interactions than elderly controls (EC). In addition, the interactions increase with disease severity from PD-FoG to PD+FoG^−^ and to PD+FoG^+^ for all intra-lobe and inter-lobe links (Fig. 2c). This overall increase is because of two factors: (i) higher levels of phase synchronization (PS) of EEG amplitude−amplitude modulations for PD patients (Fig. 2a), and (ii) brain lobe interactions are more pronounced in PD patients than in EC (Fig. 2b). The increase is consistent across all intra-lobe and inter-lobe interactions, since the PD+FoG^+^ group always yields the largest **R** × ***χ*** in the rank distribution in Fig. 4, while the EC group always yields the smallest **R** × ***χ***. Note that for the PD+FoG^+^ and the EC group, the error bars calculated by a bootstrap approach never overlap. The values for the PD-FoG and PD+FoG^−^ groups always fall in between the PD+FoG^+^ and the EC group, with the PD+FoG^−^ group generally scoring above the PD-FoG group.

Furthermore, for all groups of subjects, the interactions within the same lobe are strongest and more significant, as can be seen in Fig. 2, where the diagonal elements of all matrices show the highest values. Correspondingly, in the rank distributions in Fig. 4, the first six ranks belong to intra-lobe interactions. Inter-lobe interaction (i.e., the coupling between different lobes) is consistently weaker than intra-lobe interaction, and depends on whether lobes belong to the same brain hemisphere (higher coupling strength) or different hemispheres. For example, coupling between FML−POL (same hemisphere) is stronger than POL−POR coupling (different hemispheres) across all groups (Fig. 4).

Several EEG frequency bands have been shown to be affected by Parkinson’s disease (PD) and in particular by freezing of gait (FoG)^14–17^. Therefore, we repeated our analyses and obtained results also for other physiologically-relevant frequency bands ranging from low-frequency *θ* to high-frequency Γ waves. Comparing the brain lobe interaction networks for all five considered EEG bands in Fig. 3, we observe a very consistent pattern across all bands. In all cases, intra-lobe amplitude synchronizations (blue nodes) are weakest for the EC group and strongest for the PD+FoG^+^ group *and* inter-lobe amplitude synchronization (gray links) follow the same pattern. The differences between the PD+FoG^−^ and PD+FoG^+^ groups, i.e., PD patients with FoG symptoms who did not show and patients who did show FoG during our experiments, are most pronounced for the higher frequency bands (i.e., *β*, *γ* and Γ; Fig. 3). Particular noteworthy is the observation that for the PD+FoG^+^ group, synchronization between the frontal motor lobes (FML-FMR link) in the *γ* and Γ frequency bands becomes stronger than most other intra-hemisphere interactions (Fig. 3 and cp. Supplementary Figs. S1−S4). Overall, these results indicate that the PD-related increase in EEG amplitude synchronization occurs across all frequency bands and is directly correlated to disease severity.

**Fig. 5:**
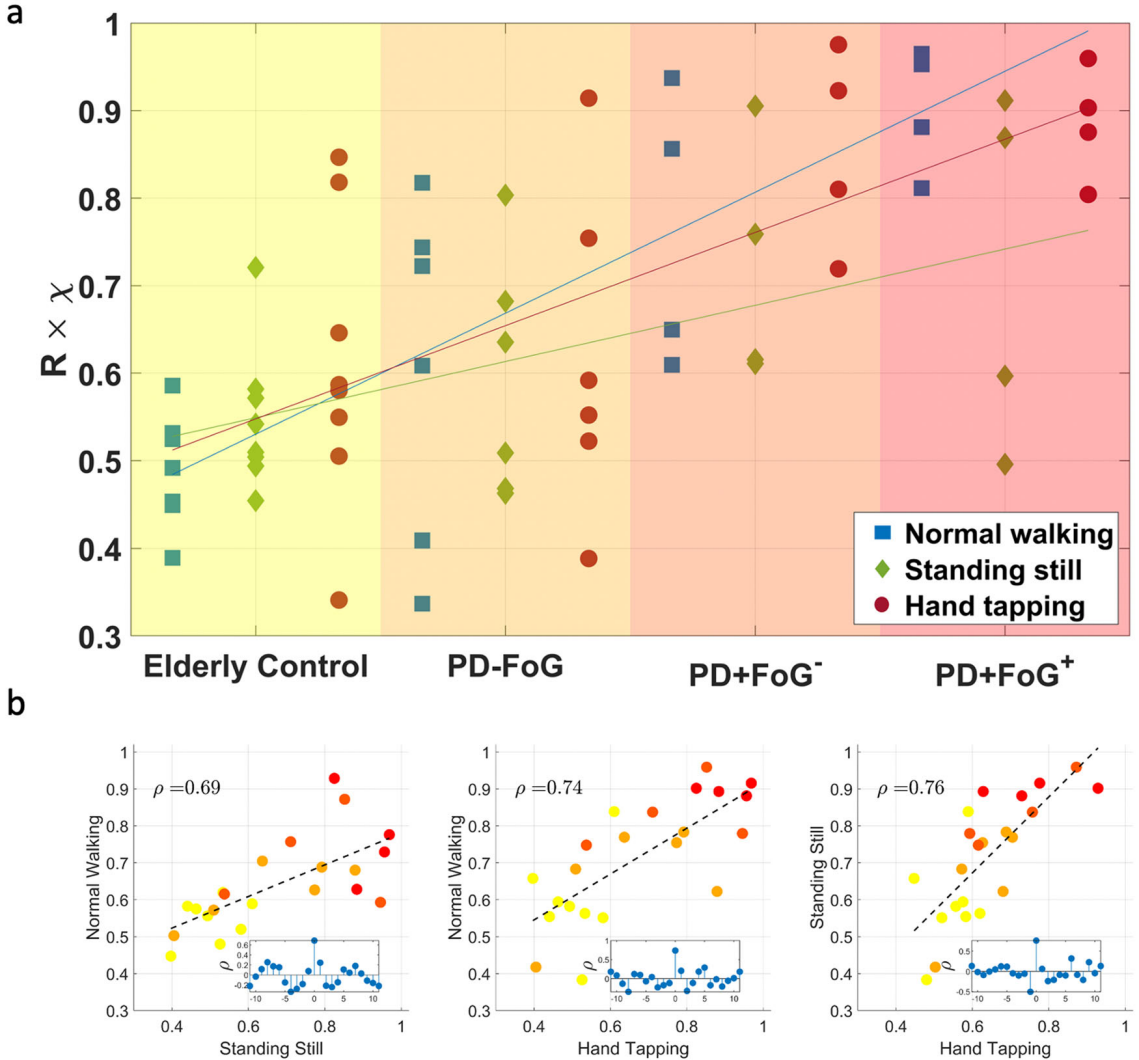
Intra-lobe brain interactions in the frontal motor lobe for the individual subjects performing different motor tasks. **a** Symbols represent the average values of FMR−FMR and FML−FML *α-*band interactions as derived from the **R** × ***χ*** matrix during normal walking (blue squares), standing still (green diamonds), and hand tapping (red circles). Values for each task are arranged in columns and each symbol represents an individual subject. The different groups are marked by different background shadings. Fitting lines highlight the trend towards higher brain lobe interactions for subjects with PD and FoG, which is seen across all three motor tasks. **b** Scatter plots show strong cross-correlations between the different motor tasks and confirm the observation of an increase of brain interactions with PD severity. The colors of the symbols correspond to the background shadings in (**a**) for patients belonging to different groups. Pearson’s correlation coefficients *ρ* are highly significant (*p* < 10^−3^) and are shown in the upper left corner of each subplot. The insets confirm the significance of the results by surrogate analysis (i.e., shuffling the number tuples of the subjects by *n* iterations, with *n* = 0 being the original un-shuffled tuple series).

The observed increase in EEG amplitude synchronization with PD is not only present during locomotion but also shows for other motor tasks. Figure 5 depicts results of intra-lobe interaction of the frontal motor lobe (FMR-FMR and FML-FML) for each individual performing the normal walking part of the experiments as well as standing still and hand tapping. There is a general trend to higher EEG synchronization values for sicker individuals and the strength of brain interactions are highly correlated between the different motor tasks.

## Discussion and conclusion

We analyzed EEG data from three PD groups with increasing levels of disease severity and one group of age-matched elderly controls. We focused on the “normal walking” state and other non-locomotor tasks and identified EEG amplitude synchronization networks of same-band frequency interactions after distinguishing between physiological and spurious synchronization. The degree of synchronization (strength of network links) in EEG amplitudes shows a dramatic increase for PD patients in more advanced stages of the disease. We also found that EEG amplitude synchronization is similar in PD-FoG and PD+FoG^−^ although both groups are generally quite different in clinical terms. This finding indicates that FoG risk can change on a daily basis where cortical areas switch between ‘prone-to-FoG’ vs. non-FoG states, and that this process could be monitored by EEG synchronization networks.

Our results are independent of EEG frequency and consistent across all studied bands from low-frequency *θ* to high-frequency Γ waves. While previous studies have reported alterations in particular brain waves in PD patients during FoG^17,44–46^, our observations of normal walking in PD+FoG patients show that EEG amplitude *synchronization* increases similarly for all frequency bands. This overall increase in EEG synchronization for advanced PD is analogous to findings in patients with Alzheimer’s disease, where increased brain activity was related to a compensation mechanism due to the process of neurodegeneration^47^. In this context, higher brain wave synchronization and increased connectivity of EEG amplitude synchronization networks may be a precursor of FoG, and our analysis method could help to monitor treatment to alleviate or eliminate FoG events thereby reducing possible injuries due to falls and improve the overall life quality of PD+FoG patients.

Parkinson’s disease is characterized by a reduction of dopaminergic neuronal input to the basal ganglia-cortical networks^48,49^. It has been shown that cortical activity as expressed in EEG signals is associated with the activity of the subthalamic nucleus (STN)^50–52^, for example, as reflected in the presence of simultaneous hyper EEG activity, specifically in the motor areas^53,54^. We hypothesize that increased inter-regional cortical phase-synchronization (PS) leading to stronger EEG coupling networks is an outcome of the variability in the subcortical input to different areas in the cortex which arises from the non-uniform impact of the dopaminergic depletion. Perhaps, as a ‘compensation’ to the non-coordinated input to the cortex, the cortical regions increase their synchronization. This hypothesis is supported by previous evidence that asymmetrical depletion of dopamine resulted in a longitudinal development of bi-cortical synchronization particularly in the motor areas^55–57^, which in the present study is expressed by FMR−FML hyper synchronization. Moreover, our hypothesis could be tested in future studies, for example, by investigating the effect of the dopaminergic medications on inter-regional network PS and by comparing EEG networks during OFF and ON conditions. If indeed confirmed, EEG network synchronization might become a clinically relevant tool to monitor and evaluate medication intake effects for PD patients. The present findings point to the possibility that persons with PD who suffer from the FoG symptom exhibit increased inter-regional network PS. However, as the pathophysiology of FoG is largely unknown^4^, it is not clear whether the potentially FoG-associated hyper synchronization is consequential or causal to the symptom. Nonetheless, the increased PS among this subgroup of persons with PD implies that a more severe disease symptomatology leads to higher values for the EEG network synchronization.

A limitation of the present study is the relatively small number of participants in each of the study groups, which limits the external validity of the present findings and warrants future confirmation with larger groups of participants. It is also important to note that in the present sample, scores on the UPDRS-Part III scale are relatively low (see Table 2), particularly for persons with PD who suffer from FoG in their OFF state (see, e.g.,^58,59^). Future research should address whether the severity of motor impairments in PD as expressed by the UPDRS-III scores are reflected by the level of inter-regional cortical PS. Distinguishing PD patients particularly through the occurrence or absence of FoG, the present results indicate that inter-regional cortical PS is higher in those who suffer from FoG as compared to those who do not. Interestingly, it appears that cortical PS can be considered as a marker of FoG intermittent risk, as those who exhibited FoG episodes during the experimental session showed higher PS as compared to freezers who were spared from the symptom in that particular time.

**Table 2:** Demographic and clinical data of the study groups.

Group	EC	PD-FoG	PD+FoG^−^	PD+FoG^+^
f/m	5/3	3/3	0/4	0/4
Age [y]	63.0 ± 8.5	69.8 ± 8.1	66.0 ± 8.3	66.6 ± 8.8
BMI	25.1 ± 3.1	26.0 ± 4.2	26.0 ± 2.3	26.5 ± 6.7
Disease duration	N/A	10.2 ± 4.7	8.0 ± 4.4	12.8 ± 5.6
L-dopa equivalent				
daily dose (LEDD) [mg]	N/A	872 ± 253	1250 ± 230	1448 ± 24
MoCA	25.8 ± 2.3	23.1 ± 5.8	21.5 ± 3.1	22.7 ± 2.2
UPDRS-Motor				
score (Part III)	N/A	15.5 ± 6.9	20.0 ± 8.2	16.5 ± 4.7

Elderly controls (EC), participants with Parkinson’s disease that do not show freezing of gait (PD-FoG), participants with Parkinson’s disease that usually have FoG but did not show it during the experiment (PD+FoG^−^), and participants with Parkinson’s disease and FoG during the experiment (PD+FoG^+^). Gender *f/m* female/male, *BMI* body mass index (in kg/m2), *MoCA* Montreal Cognitive Assessment^71^, *UPDRS* Unified Parkinson’s Disease Rating Scale^72^. Reported are means and standard deviations; the differences between the groups are not significant, except for gender, and LEDD between PD-FoG and PD+FoG^+^. We note that LEDD was not available for all subjects.

As more EEG data recorded during gait in PD patients and in particular during FoG epochs become available, future work could combine machine learning together with network analysis tools to automatically detect and perhaps even predict FoG. On the other hand, combining EEG interaction networks (high temporal resolution) with fMRI studies (high spatial resolution) can reveal important network features mapped onto brain areas that play role in freezing events. Our method of defining and analyzing EEG synchronization networks can be used as a new metric for such combined EEG-fMRI studies.

## Methods

### Data recording and preprocessing

Data from participants with Parkinson’s disease (PD) and from healthy elderly controls (EC) were recorded at the Center of Advanced Technologies in Rehabilitation (CATR) at the Chaim Sheba Medical Center at Tel HaShomer, Israel. Inclusion criteria for PD participants were: age >50 yrs, diagnosis of idiopathic PD^60^, current levodopa treatment, ability to walk unassisted and without pain for at least 100 m, being able to understand and perform verbal instructions. Exclusion criteria were: the presence of significant co-morbidities and major orthopedic problems. PD participants were examined in the OFF state, i.e., at least 12 h after the last intake of anti-Parkinsonian medication. The study protocol was approved by the Institutional Review Board (IRB) of Sheba Medical Center, and participants gave written informed consent prior to the study.

All participants performed gait trials during which they were exposed to “FoG triggers”, i.e., walking circumstances that are likely to invoke FoG among patients with PD who suffer from the FoG symptom^58^. Specifically, those conditions included (i) walking back and forth in a 12 m long and 2.5 m wide corridor performing 180^∘^ turns at the ends of the corridor, (ii) figure-eight task—continuously walking for 5 min in a figure-of-eight trajectory between two cones that were 2.5 m apart from each other, and (iii) narrow passage task—walking through a 0.5 m wide passage between cone and wall. Occasionally, the participants were instructed to stop walking (“commanded stops”) to provide a controlled condition in contrast to the unintended FoG episodes. Overall, the participants walked for 15−20 min with short breaks for rest.

Surface electroencephalogram (EEG) has been recorded by a portable system (Micromed, Mogliano Veneto, Italy) consisting of a 32-channel montage using the international 10−20 electrode placement scheme. The data were annotated by post hoc analysis of video files recorded during the gait trials. Data slices were sorted according to motion type (walking, FoG, commanded stops). To separate regular walking from FoG episodes, a set of predetermined standardized and performance-based criteria was used, as previously described^5^. For more details on FoG annotation of this database, see ref. ^22^. Additionally, after completing the walking trials, participants performed reference tasks of standing still and hand tapping with their palms by standing in front of an elevated table. For at least one minute duration each, participants engaged in alternating hand tapping and simultaneous hand tapping.

We analyzed the EEG data of four groups of subjects: (i) patients with Parkinson’s disease (PD) that never had FoG episodes (PD-FoG; *n* = 6); (ii) PD patients that usually show FoG but did not have an episode of FoG during this study (PD+FoG^–^; *n* = 4); (iii) PD patients with FoG episodes during this study (PD+FoG^+^; *n* = 4); and (iv) healthy elderly controls (EC; *n* = 8). Among the PD+FoG^+^ group, 81 FoG episodes were observed (mean ± stdev = 17.8 ± 8.1 per patient—for details on FoG episodes and triggers for each PD+FoG^+^ patient, see Table S1 in Supplementary Information). All groups are age-matched; however, subjects were not age-matched on an individual level; for demographic and clinical parameters of the study groups, see Table 2. We note that gait speed was not significantly different between the groups; however, the PD+FoG^−^ group had a significantly shorter total walking time as compared to the other groups (for more details, see Table S2 in Supplementary Information).

EEG data were preprocessed using EEGLAB^61^. For each gait task and each participant, data preprocessing steps included: (i) omitting data from electrodes with high impedance (>10 kΩ) and high standard deviation^62^; (ii) data down-sampling from 2048 to 256 Hz; (iii) basic finite impulse response high-pass filtering with a threshold of 0.1 Hz; (iv) applying an independent component analysis (ICA) (‘runica’ implementation)^63^ for the removal of eye movements and general movement artifacts. The ICA algorithm exploits the fact that several EEG electrodes are affected by the same artifacts, in particular movement artifacts. This common source is identified by the algorithm, and its relative contribution to each electrode is subtracted. Using component activation, power spectra, and maps, the different components were visually inspected, and a minimal number of components (2 or 3) has been removed. We have developed this pre-processing pipeline prior to this study while investigating how different kinds of movements and gait speeds affect EEG, which artifact types are encountered, and what techniques could be used to ‘clean’ EEG data without removing relevant physiological information (for more details, see ref. ^31^). In agreement with earlier studies^30,64,65^, we found that the regular ICA approach (“runica” or “AMICA”) is sufficient and appropriate for movement artifact removal in EEG data for normal walking speeds and even light jogging (at speed 2.2 m/s). A comparison with other methods of EEG artifact removal (e.g., Automatic subspace reconstruction (ASR)^66^ yielded comparable results.

The preprocessed EEG data for each channel have been used to extract characteristic “brain wave” signals via bandpass filtering in the following six frequency bands: *δ* [0.5−3.99 Hz], *θ* [4−7.79 Hz], *α* [7.8−15.59 Hz], *β* [15.6−31.19 Hz], low and high gamma with *γ* [40−62.39 Hz] and Γ [62.4−90 Hz], respectively. We obtain a total of 192 signals *S*^*j*^(*t*), *j* = 1, …, 192, six frequency-component signals for 32 EEG channels. Signals from the four midline electrodes (Fz, Cz, Pz, Oz)), as well as the mastoid electrodes M1 and M2, were excluded from the following analysis (see Fig. 1a).

### Phase synchronization of EEG amplitudes

**Fig. 6:**
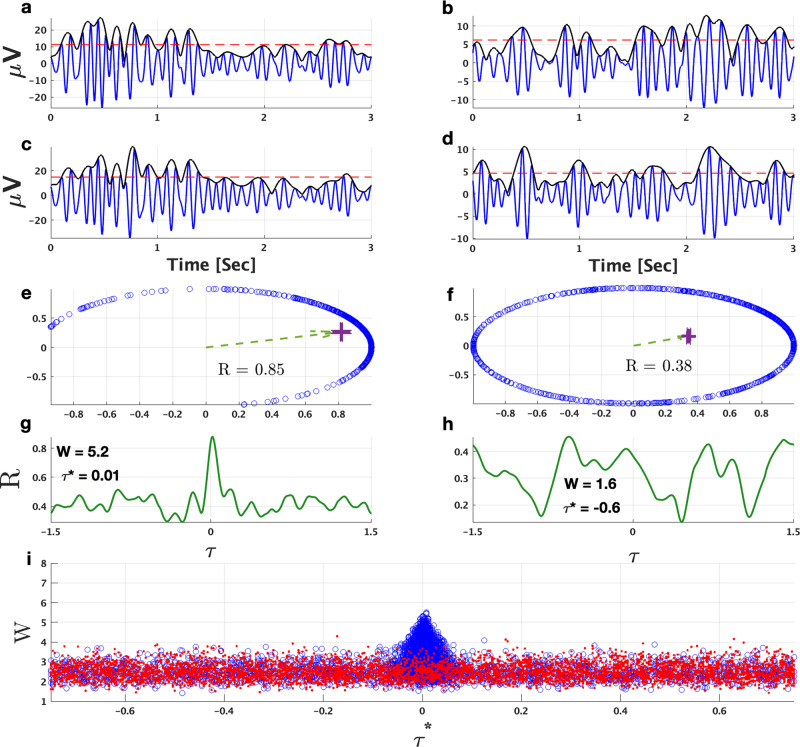
Phase synchronization of amplitude-amplitude modulations and surrogate analysis to identify significant interactions. Two pairs of *α* frequency-band signals (blue curves in (**a**)−(**c**) and (**b**)−(**d**)) from different EEG electrodes were obtained by applying a [7.8−15.59 Hz] bandpass-filter to the preprocessed EEG data. The black curves in each of these panels are the corresponding instantaneous amplitudes calculated by the analytic signal approach, Eq. (1). Red dashed lines are the corresponding averages ${\langle {A}^{j}(t)\rangle }_{L}$ subtracted when applying the analytic signal approach to derive phases of these instantaneous amplitudes. **e** Phase differences of the instantaneous amplitudes of (**a**)−(**c**) are clustered on the unit circle leading to a high synchronization index of *R* = 0.85 (Eq. (2)). In contrast, the signals in (**b**)−(**d**) are less synchronized as can be seen in (**f**), where the corresponding phase differences are distributed on the unit circle yielding a low index of *R* = 0.38. **g**, **h** Phase synchronization index *R* as a function of the shift *τ* between the instantaneous amplitude signals (**a**) vs. (**c**) and (**b**) vs. (**d**), respectively. The phase synchronized amplitude signals from (**a**) and (**c**) yield a maximum *R* at shift ${\tau }^{* }={\left.\tau \right|}_{R(\tau )\equiv {R}_{\max }}^{}=0$, and *R*(*τ*) decays rapidly for ∣*τ*∣ > 0. For the much lower synchronized signals from (**b**) and (**d**), however, *R*(*τ*) shows fluctuating behavior without clear decay. A significance value *W* characterizes *R*(*τ*) by normalizing ${R}_{\max }$ by the mean and standard deviation of *R*(*τ*) (Eq. (3)). Correspondingly, we obtain a higher *W* value for panel (**g**) (*W* = 5.2) than for panel (**h**) (*W* = 1.6). We utilize *W* to characterize the *significance* of the interaction between two signals. Panel (**i**) indicates that the highest *W* values are observed for τ^*^ ≈ 0. In this scatter plot we show 1000 *α*−*α* samples of *W* vs. τ^*^ for real data (blue circles) and surrogate data (red dots). Real signals are taken from the same patient (using different EEG electrodes), whereas surrogate pairs were chosen randomly from different patients. Clearly, higher *W* values are obtained for real signals for τ^*^ ≈ 0. The surrogate analysis does not lead to high *W* values around τ^*^ ≈ 0 and shows a uniform *W* vs. τ^*^ distribution.

In the first step of our data analysis procedure based on^41^, for each signal *S*^*j*^(*t*), we construct the analytic signal *ξ*(*t*)^67,68^ by

1 $$\xi (t)={S\,}^{j}(t)+{{{{{\mathrm{i}}}}}}{{\widetilde{S\,}}}^{j}(t)={A}^{j}(t)\exp [{{{{{\mathrm{i}}}}}}{\varphi \,}^{j}(t)],$$

in order to obtain the instantaneous amplitude *A*^*j*^(*t*) and instantaneous phase *φ*^*j*^(*t*) (*i* = imaginary unit; ${{\widetilde{S}}}^{j}(t)$ is the Hilbert transform of *S*^*j*^(*t*)). Then, by constructing the analytic signal of ${A}^{j}(t)-{\langle {A}^{j}(t)\rangle }_{L}$, we obtain the instantaneous phase *ϕ*^*j*^(*t*) of the amplitude signal (cp. Fig. 6a−d). Here, ${\langle {A}^{j}(t)\rangle }_{L}$ denotes the average over time windows of *L* seconds. We note that the length *L* of each segment is chosen according to the analyzed frequency band in order to ensure that about 10−15 amplitude oscillations are present in each segment in order to obtain meaningful synchronization results (see Table 1 and Fig. 6a−d). Based on this consideration, we removed *δ-*band interactions from the following analyses because of insufficient statistics (<100 available segments). We also note that for the subsequent analyses we select only windows recorded during normal walking that do not contain stops, FoG episodes, or FoG triggers.

We quantify phase synchronization in the amplitude-amplitude modulations for different signals *j*_1_ and *j*_2_, by calculating the phase differences ${\phi }^{{j}_{1}}(t)-{\phi }^{{j}_{2}}(t)$ and average their complex exponentials over segments *ν* of length *L* to obtain the synchronization index^33^

2 $${R}^{{j}_{1},{j}_{2}}(\nu )=| {\langle \exp [{{{{{\mathrm{i}}}}}}\left(\right.{\phi }^{{j}_{1}}(t)-{\phi }^{{j}_{2}}(t)]\rangle }_{\nu }| .$$

In general, *R* will be small (closer to 0) if the two amplitude signals are not phase-synchronized, i.e., their phase differences are random and their complex exponentials do not show clustering on the unit circle. In case of a consistent phase synchronization of the signals’ amplitudes (“amplitude cross-modulation”^41^), *R* will have values closer to 1 (cp. Fig. 6e, f). We calculate the synchronization index *R* between signals of the same frequency stemming from different EEG electrodes.

### Probing significant interactions in amplitude synchronization

In order to distinguish significant from non-significant interactions between signals *j*_1_ and *j*_2_, we study how their amplitude-phase synchronization index *R* decays when shifting the signals against each other. Panels (g) and (h) in Fig. 6 show examples of *R* versus the time shift *τ* and suggest that more synchronized signals (with higher *R* values) have a marked decay of *R*(*τ*) (panel (g)) that may not be seen for less synchronized signals (panel (h)). To quantify this observation, we define a *significance* value *W* that normalizes the maximum phase synchronization index ${R}_{\max }$ (detected at a particular time shift τ^*^) by the “background noise” characterized by the mean and standard deviation of *R*(*τ*). Colloquially, *W* gives an estimate of how much ${R}_{\max }$ “stands out” from the noise background. It can be defined by

3 $$W=\frac{{R}_{\max }-\langle R(\tau )\rangle }{\sigma (R(\tau ))},$$

where 〈*R*(*τ*)〉 (*σ*(*R*(*τ*))) is the mean (standard deviation) of *R*(*τ*) in the time window *τ* ∈ [−*L*/2, *L*/2]. From Eq. (3) one can see that *W* quantifies how many standard deviations ${R}_{\max }$ is above noise level. Thus, the larger ${R}_{\max }$ is compared to the background, the higher is *W*, indicating a more significant coupling between signals *j*_1_ and *j*_2_. On the other hand, if ${R}_{\max }$ does not stand out from the *R*(*τ*) background, *W* is low implying a non-significant *j*_1_ − *j*_2_ interaction. Fig. 6i shows results obtained from 1000 pairs of *α*-amplitude signals, where each pair of signals is either taken from the same subject (“real” data) or from different subjects (“surrogate” data). It can be seen from the figure that high *W* values obtained for the real data are usually detected for small shifts τ^*^, which is consistent with the fact that brain waves generally propagate rather quickly^69^. However, for the surrogate data, there is no particular clustering of high *W* values at any τ^*^, and *W* vs. τ^*^ is uniformly distributed. Thus, we consider *j*_1_–*j*_2_ interactions only as significant if τ^*^ ∈ [−0.05, 0.05] seconds and *W* > 2.5. These values were chosen so that only about 1.5% of the surrogate data fulfill this condition.

### Bootstrapping approach

Bootstrapping is a resampling method to determine measures of accuracy (e.g., variance, standard error, confidence intervals) by calculating estimators of the underlying distribution function from the data sample^70^. In particular, random sampling with replacement is used to simulate the sampling process. For Fig. 4, we calculate the error bars by the following procedure: out of all values of a particular matrix element (as shown in Fig. 2c), a new set of values is randomly drawn and for each sample the unweighted mean is calculated. This process is repeated 100 times and the standard deviation of the obtained means is an estimate for the standard error^70^.

### Reporting summary

Further information on research design is available in the Nature Research Reporting Summary linked to this article.

## Supplementary information

Supplementary Information

Reporting Summary

## Data Availability

We utilize de-identified multi-channel EEG recordings that were obtained from participants with Parkinson’s disease (PD) and from healthy elderly controls (EC) at the Center of Advanced Technologies in Rehabilitation (CATR) at the Chaim Sheba Medical Center at Tel HaShomer, Israel. Data can be obtained upon reasonable request by contacting  the corresponding authors.

## Code availability

For data analyses standard MATLAB subroutines (spectral power, window averaging, phase synchronization analysis) are used in a particular order as explained in the Methods section of the paper.
