# Supplementary material for: Connectivity of EEG synchronization networks increases for Parkinson’s disease patients with freezing of gait
Source: Commun Biol. 2021 Aug 30;4:1017. doi: 10.1038/s42003-021-02544-w (PMC8405655; doi:10.1038/s42003-021-02544-w)
Supplement: Supplementary file 1 — Supplementary Information [file 42003_2021_2544_MOESM1_ESM.pdf]

Supplementary Information

	MC-P05	MC-P22	MC-P20	MC-P08
Start hesitation	-	-	7	1
Turn hesitation	-	9	4	31
Hesitation in tight quarters	11	18	-	-
Total number FoG	11	27	11	32
Mean duration [s]	5.2	4.1	7.0	2.9

Supplementary Table S1: Number of FoG episodes, triggers and mean durations for each of the PD+FoG$^+$ patients.

Group	EC	PD-FoG	PD+FoG$^-$	PD+FoG$^+$
Gait speed [m/s]	0.90 ± 0.17	0.74 ± 0.11	0.75 ± 0.15	0.81 ± 0.29
Analyzed total walking time [s]	356 ± 92	317 ± 64	206 ± 39	347 ± 111

Supplementary Table S2: Means and standard deviations for the respective groups. While gait speed was not significantly different between the groups, the PD+FoG$^-$ group had a significantly shorter total walking time as compared to all other groups.

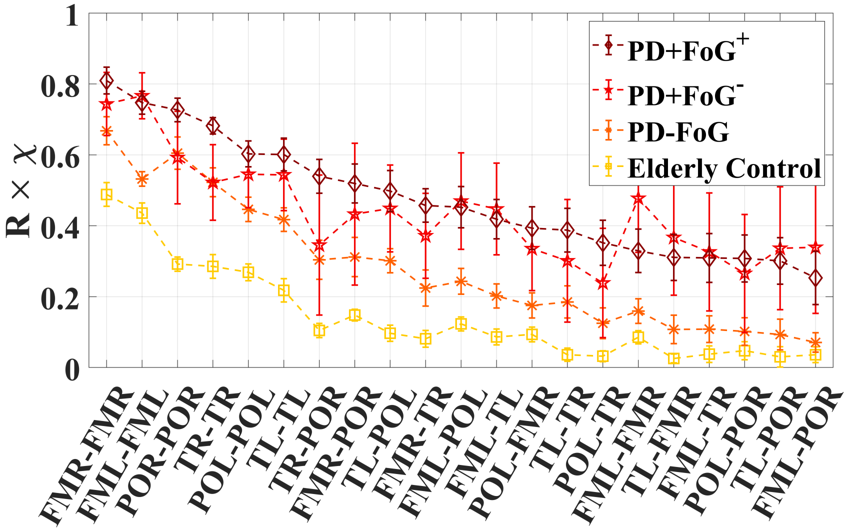

Supplementary Figure S1

Supplementary Fig. S1 caption: **Rank distributions for the strength of brain lobe interactions for θ−θ coupling.** Group-averaged values of individual brain lobe θ−θ interactions for the different groups of subjects. Ranking follows the values of the PD+FoG$^+$ group. Ranks 1 and 2 correspond to interactions within the frontal motor areas (FMR-FMR and FML-FML) that are strongest for all groups. Note that values of each $\mathbf{R} \times \chi$ matrix element are consistently highest for PD+FoG groups and lowest for EC, with PD-FoG falling in-between. Symbols and error bars represent the group means and standard error, respectively. Error bars have been calculated using a bootstrap method [70]. The high synchronization strength observed for the PD+FoG$^+$ group is seen across all bands, and the difference between PD+FoG$^+$ to all other groups increases with higher frequencies (cp. all supplementary figures). Note that the highly fluctuating ranking curve for $\theta - \theta$ coupling for the PD+FoG$^-$ group is due to relatively low number of available data for this frequency band and patient group.

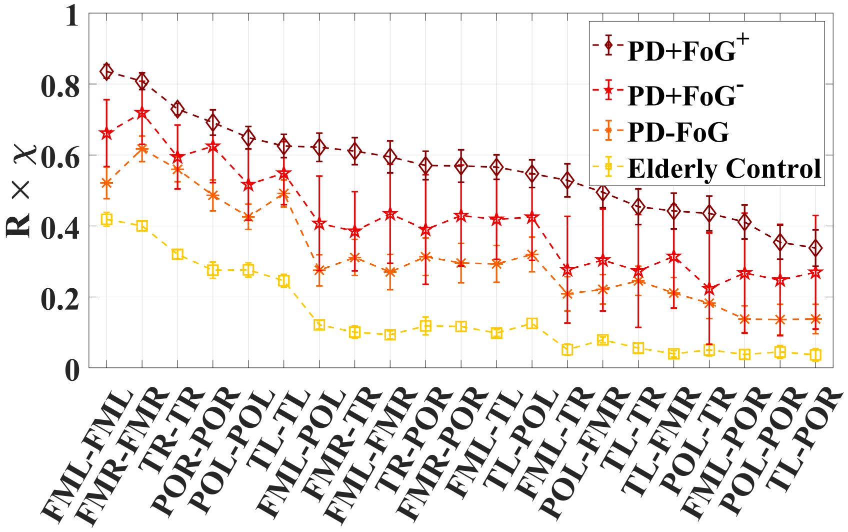

Supplementary Figure S2

Supplementary Fig. S2 caption: **Rank distributions for the strength of brain lobe interactions for $\beta - \beta$ coupling.** Group-averaged values of individual brain lobe $\beta - \beta$ interactions for the different groups of subjects. Ranking follows the values of the PD+FoG+ group. Ranks 1 and 2 correspond to interactions within the frontal motor areas (FMR-FMR and FML-FML) that are strongest for all groups. Note that values of each $\mathbf{R} \times \chi$ matrix element are consistently highest for PD+FoG+ and lowest for EC, with PD-FoG and PD+FoG- falling in-between. Symbols and error bars represent the group means and standard error, respectively. Error bars have been calculated using a bootstrap method [70].

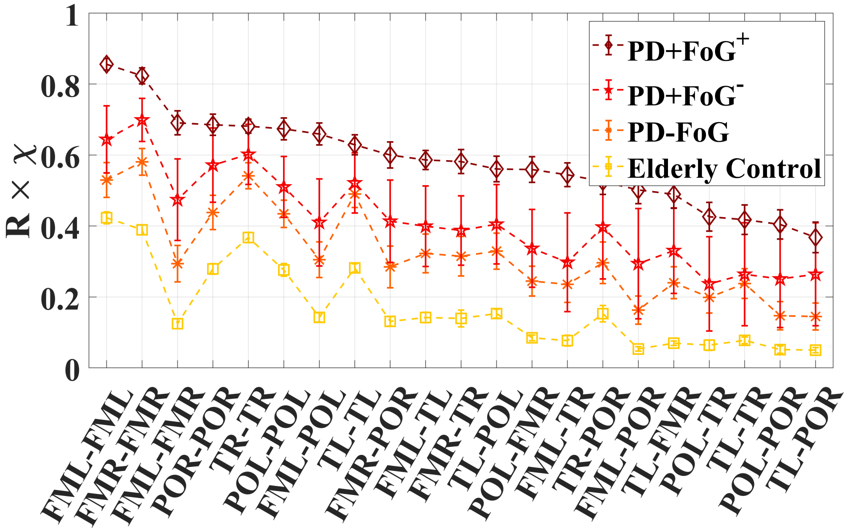

Supplementary Figure S3

Supplementary Fig. S3 caption: **Rank distributions for the strength of brain lobe interactions for $\gamma - \gamma$ coupling.** Group-averaged values of individual brain lobe $\gamma - \gamma$ interactions for the different groups of subjects. Ranking follows the values of the PD+FoG$^+$ group. Ranks 1 and 2 correspond to interactions within the frontal motor areas (FMR-FMR and FML-FML) that are strongest for all groups. Note that values of each $\mathbf{R} \times \chi$ matrix element are consistently highest for PD+FoG$^+$ and lowest for EC, with PD-FoG and PD+FoG$^-$ falling in-between. Symbols and error bars represent the group means and standard error, respectively. Error bars have been calculated using a bootstrap method [70].

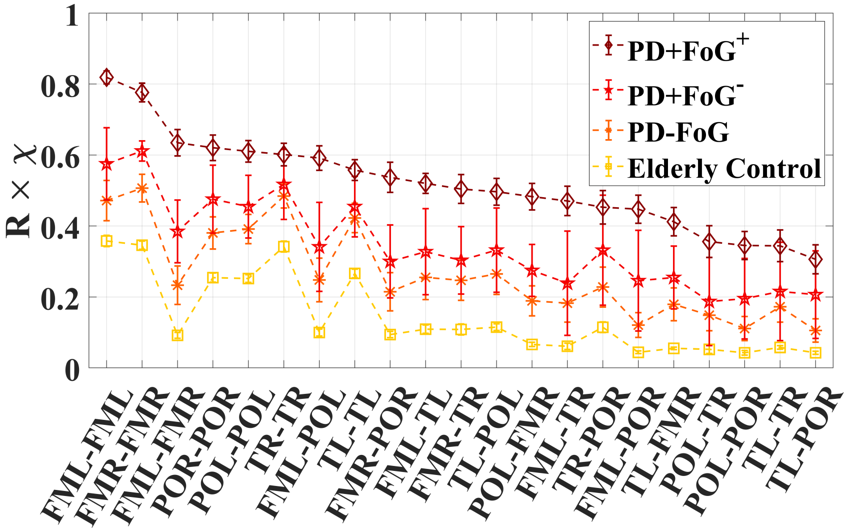

Supplementary Figure S4

Supplementary Fig. S4 caption: **Rank distributions for the strength of brain lobe interactions for $\Gamma - \Gamma$ coupling.** Group-averaged values of individual brain lobe $\Gamma - \Gamma$ interactions for the different groups of subjects. Ranking follows the values of the PD+FoG^{+} group. Ranks 1 and 2 correspond to interactions within the frontal motor areas (FMR-FMR and FML-FML) that are strongest for all groups. Note that values of each $\mathbf{R} \times \chi$ matrix element are consistently highest for PD+FoG^{+} and lowest for EC, with PD-FoG and PD+FoG^{-} falling in-between. Symbols and error bars represent the group means and standard error, respectively. Error bars have been calculated using a bootstrap method [70].
