# Supplementary material for: Connectivity of EEG synchronization networks increases for Parkinson’s disease patients with freezing of gait
Source: Commun Biol. 2021 Aug 30;4:1017. doi: 10.1038/s42003-021-02544-w (PMC8405655; doi:10.1038/s42003-021-02544-w)
Supplement: Supplementary file 2 — Reporting Summary [file 42003_2021_2544_MOESM2_ESM.pdf]

nature research

Corresponding author(s): Ronny P. Bartsch

Last updated by author(s): Jan 17, 2021

Reporting Summary

Nature Research wishes to improve the reproducibility of the work that we publish. This form provides structure for consistency and transparency in reporting. For further information on Nature Research policies, see our Editorial Policies and the Editorial Policy Checklist.

Statistics

For all statistical analyses, confirm that the following items are present in the figure legend, table legend, main text, or Methods section.

n/a	Confirmed	
☐	☒	The exact sample size (*n*) for each experimental group/condition, given as a discrete number and unit of measurement
☐	☒	A statement on whether measurements were taken from distinct samples or whether the same sample was measured repeatedly
☐	☒	The statistical test(s) used AND whether they are one- or two-sided<br>*Only common tests should be described solely by name; describe more complex techniques in the Methods section.*
☒	☐	A description of all covariates tested
☒	☐	A description of any assumptions or corrections, such as tests of normality and adjustment for multiple comparisons
☐	☒	A full description of the statistical parameters including central tendency (e.g. means) or other basic estimates (e.g. regression coefficient) AND variation (e.g. standard deviation) or associated estimates of uncertainty (e.g. confidence intervals)
☒	☐	For null hypothesis testing, the test statistic (e.g. *F*, *t*, *r*) with confidence intervals, effect sizes, degrees of freedom and *P* value noted<br>*Give P values as exact values whenever suitable.*
☒	☐	For Bayesian analysis, information on the choice of priors and Markov chain Monte Carlo settings
☒	☐	For hierarchical and complex designs, identification of the appropriate level for tests and full reporting of outcomes
☒	☐	Estimates of effect sizes (e.g. Cohen's *d*, Pearson's *r*), indicating how they were calculated

Our web collection on statistics for biologists contains articles on many of the points above.

Software and code

Policy information about availability of computer code

Data collection	We utilize de-identified multi-channel EEG recordings that were obtained from participants with Parkinson's disease (PD) and from healthy elderly controls (EC) at the Center of Advanced Technologies in Rehabilitation (CATR) at the Chaim Sheba Medical Center, Tel HaShomer, Israel.
Data analysis	For data analyses standard MATLAB subroutines (spectral power, window averaging, phase synchronization analysis) are used in particular order as explained in the Methods section of the manuscript.

For manuscripts utilizing custom algorithms or software that are central to the research but not yet described in published literature, software must be made available to editors and reviewers. We strongly encourage code deposition in a community repository (e.g. GitHub). See the Nature Research guidelines for submitting code & software for further information.

Data

Policy information about availability of data

All manuscripts must include a data availability statement. This statement should provide the following information, where applicable:

- Accession codes, unique identifiers, or web links for publicly available datasets
- A list of figures that have associated raw data
- A description of any restrictions on data availability

Data can be obtained upon request by contacting Dr. Meir Plotnik (Meir.PlotnikPeleg@sheba.health.gov.il).

Field-specific reporting

Please select the one below that is the best fit for your research. If you are not sure, read the appropriate sections before making your selection.

☒ Life sciences ☐ Behavioural & social sciences ☐ Ecological, evolutionary & environmental sciences

For a reference copy of the document with all sections, see nature.com/documents/nr-reporting-summary-flat.pdf

Life sciences study design

All studies must disclose on these points even when the disclosure is negative.

Sample size	- multi-channel EEG recordings are obtained from 11 participants with Parkinson's disease and 8 healthy elderly controls performing over ground walking experiments in a gait laboratory
Data exclusions	- no data were excluded from the analysis
Replication	- not relevant for our study
Randomization	- not relevant for our study
Blinding	- not relevant for our study

Reporting for specific materials, systems and methods

We require information from authors about some types of materials, experimental systems and methods used in many studies. Here, indicate whether each material, system or method listed is relevant to your study. If you are not sure if a list item applies to your research, read the appropriate section before selecting a response.

Materials & experimental systems

n/a	Involved in the study	
☒	☐	Antibodies
☒	☐	Eukaryotic cell lines
☒	☐	Palaeontology and archaeology
☒	☐	Animals and other organisms
☐	☒	Human research participants
☒	☐	Clinical data
☒	☐	Dual use research of concern

Methods

n/a	Involved in the study	
☒	☐	ChIP-seq
☒	☐	Flow cytometry
☒	☐	MRI-based neuroimaging

Human research participants

Policy information about studies involving human research participants

Population characteristics	11 participants with Parkinson's disease (mean age: 67yrs) and 8 healthy elderly controls (mean age: 63 yrs).
Recruitment	Participants with Parkinson's disease and healthy elderly controls were recruited from the Movement Disorders Institute at Sheba Medical Center (for details on inclusion and exclusion criteria, see Data and Methods section of the manuscript). No potential bias was identified.
Ethics oversight	The study protocol was approved by the Institutional Review Board (IRB) of Sheba Medical Center, Israel

Note that full information on the approval of the study protocol must also be provided in the manuscript.
